# Editorial: Confronting antimicrobial resistance: trends, interventions, and socio-economic impacts

**DOI:** 10.3389/fpubh.2026.1939391

**Published:** 2026-09-07

**Authors:** Daniel Diaz, Corina Diana Ceapǎ

**Affiliations:** 1 Public Health Intelligence Unit, National Institute of Public Health, Cuernavaca, Morelos, Mexico; 2 Microbiology Laboratory, Department of Natural Products Chemistry, Institute of Chemistry, National Autonomous University of Mexico (UNAM), Coyoacan, Mexico

**Keywords:** antimicrobial resistance (AMR), antimicrobial stewardship, genomic surveillance, infection prevention and control, socio-economic determinants

Antimicrobial resistance (AMR) is a silent threat, deeply shaping the present, and future of human society. It is a multifaceted crisis that touches public health, agriculture, communities, and global supply chains. As shown by studies in this Topic—from high MRSA burdens in Rwanda (Rutare et al.) to cross-species ESBL-producing *Klebsiella pneumoniae* in Peruvian farms (Benavides et al.)—AMR is not a distant risk but an active, measurable force shaping clinical outcomes and community health.

Across the contributions in this Research Topic, readers will find clear evidence that antimicrobial resistance is shaped simultaneously by community behaviors, clinical decision-making, environmental pathways, and the governance structures that enable or constrain stewardship. Together, these studies illustrate how AMR emerges from everyday practices—in households, clinics, farms, ICUs, and markets—and how targeted, context-specific interventions can meaningfully bend the curve.

**Surveillance must be extended and made actionable**. Surveillance emerges as a cornerstone of AMR control. Facility-level detection (Kiggundu et al.), routine ICU screening (Zhang H. et al.), and genomic analyses (Rutare et al.; Sanmak et al.) fill distinct gaps in early detection. Whole-genome sequencing (WGS) transforms descriptive surveillance into operational intelligence by identifying transmission clusters, mobile genetic elements, and high-risk clones. This is evident in the pandemic-era diversification of *Acinetobacter baumannii* in Mexico (Alejo et al.) and the circulation of ESBL-producing *Klebsiella pneumoniae* across humans, livestock, dogs, and water in Peruvian farms (Benavides et al.). Timeliness is critical. Tiered detection—where culture-based alerts trigger genomic follow-up—allows WGS to refine transmission maps and reveal plasmid-borne resistance capable of crossing species and settings. Effective surveillance also depends on rapid data flow between laboratories, IPC teams, clinicians, and public health authorities, requiring interoperable reporting systems and analytic capacity. These insights reinforce that genomic surveillance must be embedded within One Health frameworks to be meaningful.

**One Health is key**. A One Health lens is essential for understanding and mitigating AMR. Resistant organisms and resistance genes circulate across humans, animals, and environmental reservoirs, driven by agricultural antibiotic use, informal veterinary dispensing, and wastewater contamination. Integrated sampling that includes animal, farm, and wastewater sources consistently reveals hidden reservoirs and transmission pathways that clinical data alone miss. Farm-level genomic work in Peru (Benavides et al.) and socio-ecological analyses in lakeshore communities in the Philippines (Dalisay et al.) illustrate how food systems, water pathways, and livestock practices contribute to AMR spread. Regulatory alignment is equally critical: veterinary and agricultural antibiotic policies must be harmonized with human stewardship to prevent farm-level selection pressure from undermining hospital-based gains. Market-level interventions, including vendor training and enforcement of prescription-only rules, show measurable reductions in inappropriate antimicrobial access (Dambazau et al.).

**Knowledge alone is not enough**. Knowledge alone rarely translates into safe antibiotic practices. Studies among pharmacy students (Maqbool et al.) and animal drug vendors (Dambazau et al.) reveal persistent gaps between awareness and practice. Community-level research highlights problematic household use, storage, and disposal of antibiotics in settings lacking return or take-back programs. In contrast, strong WASH infrastructure in rural South India is associated with markedly lower antimicrobial consumption, with all reported use being prescription-based (Mathew et al.). Effective stewardship requires hands-on, rotation-embedded training; structured engagement with human and animal drug vendors; and community entry points such as hygiene initiatives and school-based programs. School-based AMR education in Iraq significantly improved teachers' knowledge and attitudes (Khudhur et al.), demonstrating the value of educators as multipliers of community awareness.

**ICUs and farms are priority settings**. High-risk clinical environments—including ICUs, surgical wards, and high-risk procedural pathways—consistently act as amplification points for resistant organisms. ICU isolates show markedly higher resistance burdens (Munyemana et al.), and bloodstream infections caused by carbapenem-resistant Gram-negative bacilli carry extremely high mortality when empirical therapy is ineffective (Corona-Nakamura et al.). Active ICU screening for CRE fecal carriage reduces onward transmission (Zhang H. et al.), while ward-stratified and specimen-specific antibiograms improve empiric therapy accuracy. Stewardship interventions such as antimicrobial timeouts and prospective audit-and-feedback consistently decrease unnecessary broad-spectrum exposure. Ineffective empirical therapy remains an independent predictor of mortality in MDR Gram-negative bloodstream infections (Corona-Nakamura et al.), underscoring the lifesaving importance of rapid diagnostics and locally tailored empiric protocols. Agricultural settings also function as amplification points. The detection of identical ESBL-producing *K. pneumoniae* clones across humans, animals, and water sources (Benavides et al.) and the socio-ecological drivers identified in lakeshore communities (Dalisay et al.) highlight the need for integrated farm sanitation, regulated veterinary supply chains, and environmental monitoring.

**Genomics informs action**. Policy, governance, and financing consistently appear as structural determinants of AMR control, with multiple studies documenting persistent gaps such as fragmented surveillance, uneven laboratory capacity, and weak regulatory enforcement. Addressing these weaknesses requires political will, sustained financing, and coordinated action across sectors, supported by integrated surveillance networks linking hospital, veterinary, and environmental laboratories; regulatory modernization that strengthens prescription-only enforcement while safeguarding access to essential medicines; predictable budgets for sequencing, rapid diagnostics, stewardship teams, and WASH infrastructure; and administrative multidisciplinary teams that reduce unnecessary perioperative prophylaxis (Li Y. et al.) and help identify risk factors for extended prophylaxis beyond 24 h (Li Z. et al.). Locally adapted stewardship indicators, such as the AWaRe-based metrics developed for South African primary care (Chigome et al.), further illustrates how governance can translate into practical improvements. Yet important gaps remain—referral bias in sequencing programs, undersampled environmental reservoirs, and limited economic analyses of AMR burden in resource-constrained settings—while Zaheer highlights the substantial socioeconomic impacts of AMR in Pakistan, where rising resistance, fragmented surveillance, widespread non-prescription use, and financial strain on households and health systems underscore the urgency of coordinated, well-financed national responses.

To appreciate how these systemic issues translate into real-world outcomes, the main conclusions of each article provide a detailed view of the mechanisms driving AMR across clinical, community, and environmental contexts.

Kiggundu et al. describe how AMR remains a “silent pandemic” because global outbreak reporting systems rarely highlight resistant-pathogen events. They propose adapting WHO Disease Outbreak News workflows to include standardized AMR reporting, tiered alert thresholds, and stronger links between facility, national, and international response mechanisms—a clear operational case for early, visible reporting that mobilizes IPC and AMS resources where they matter most.

Maqbool et al. surveyed final-year pharmacy students in Karachi and found strong knowledge and attitudes about antifungal resistance but weaker translation into practice. They highlight an education-to-practice gap and recommend targeted curricular changes and hands-on antifungal stewardship training, especially in contexts where over-the-counter access persists.

Rutare et al. report a high MRSA burden (43% of *S. aureus* isolates) at a Rwandan tertiary hospital, with MRSA linked to increased mortality and limited first-line options—underscoring the need for ICU-focused infection control and stewardship. Complementing this, Sanmak et al. analyzed 5 years of tracheal aspirate cultures in Türkiye and found Gram-negative pathogens dominating ventilator-associated pneumonias, with higher resistance in ICU isolates and a notable temporal shift around 2020. Together, these studies show that critical-care settings are AMR hotspots where rapid diagnostics, tailored empiric therapy, and strict IPC can save lives.

Benavides et al. used whole-genome sequencing to show that ESBL-producing *Klebsiella pneumoniae* circulates across humans, livestock, dogs, and water on Peruvian farms, with near-identical clones found in different hosts—clear evidence of cross-species transmission and environmental contamination. Alejo et al. examined *Acinetobacter baumannii* genomes in Mexico and documented pandemic-era diversification: more resistance genes, enriched mobile elements, and shifts in virulence and metabolic traits. These genomic studies demonstrate how environmental reservoirs and mobile genetic elements drive AMR spread and why surveillance must cross human, animal, and environmental boundaries.

Dambazau et al. surveyed animal drug vendors in Kano State, Nigeria, finding that only a minority had adequate AMR knowledge or good dispensing practices despite generally positive attitudes. Education and prior training improved knowledge, but poor practices persisted, pointing to the need for combined strategies: vendor training, enforcement of prescription rules, and market-level incentives to reduce over-the-counter antimicrobial sales.

Community-level determinants of antimicrobial use remain central. Mathew et al. show how strong WASH infrastructure in rural Kerala, India directly reduces antimicrobial consumption: only 15.5% of households reported antimicrobial use in the previous year, and all use was prescription-based. Their findings reinforce that hygiene access is not merely a development goal but a practical AMR lever, reducing demand for antibiotics at the community level.

Jamil et al. present a structured, institution-wide approach to developing evidence-based guidelines for diabetic foot infections, demonstrating how integrating routine clinical data, microbiology trends, and local resource constraints can transform fragmented practice into coherent, context-appropriate protocols.

Hospital-based studies highlight the life-or-death consequences of delayed or ineffective therapy. Corona-Nakamura et al. report that bloodstream infections caused by carbapenem-resistant Gram-negative bacilli in Mexico carry extremely high mortality, especially when empirical therapy fails. Their striking result—ineffective empirical treatment increased mortality more than tenfold (HR 10.2)—underscores the urgency of rapid diagnostics and locally informed empiric guidelines. Similarly, Munyemana et al. document a 21.9% MDR prevalence at a Rwandan referral hospital, with MDR infections linked to longer hospital stays and 22% mortality, reinforcing the need for robust IPC and stewardship in LMIC tertiary centers.

Outpatient stewardship also emerges as a critical intervention point. Vats et al. argue that “the most critical intervention point for AMS is when a prescription is issued,” mapping gaps in Abu Dhabi's outpatient systems—uneven surveillance, fragmented e-health access, and under-represented clinics—and proposing a six-pillar framework that integrates system design, technology, pharmacy engagement, and community participation.

Several studies emphasize the value of adapting global guidance to local realities. Chigome et al. demonstrates how South Africa's primary care experts expanded the AWaRe-based indicator set into seven clinically relevant categories, showing that local adaptation is essential for feasible, implementable stewardship metrics in LMICs.

Community engagement appears repeatedly as a powerful but underused tool. Mishra et al. argue that participatory approaches—citizen science, youth ambassadors, and civil-society partnerships—generate locally relevant evidence and strengthen national action plans. Their central insight, that “community engagement can improve AMR outcomes by generating a deeper understanding of the unique and complex community drivers of AMR,” resonates across multiple country contexts.

Critical-care environments remain hotspots for transmission and amplification. Zhang H. et al. report 12.6% fecal colonization with CRE in Shanghai ICUs, dominated by *Klebsiella pneumoniae* ST11-KL64 carrying *blaKPC-2*. Active screening combined with IPC interventions reduced CRE infections and revealed clonal spread between patients and the environment—a clear demonstration of how routine ICU surveillance can interrupt transmission. Complementing this, Ao et al. present a case where broad-spectrum empiric therapy for suspected necrotizing fasciitis ultimately produced antimicrobial-associated drug fever, reminding clinicians that stewardship principles must remain active even in high-risk surgical settings.

Governance and accountability also shape antimicrobial use. Li Y. et al. show that an administrative multidisciplinary team overseeing perioperative prophylaxis reduced antibiotic use for clean (Class I) incisions from 33.7% to 25.9% without increasing SSI rates—a scalable model for hospitals seeking to curb unnecessary prophylaxis. Another study by Li Z. et al. finds that 12.6% of pancreatic surgery patients received prophylaxis beyond 24 h, driven largely by procedures performed in non-specialized centers, highlighting organizational targets for stewardship.

Environmental and socio-ecological drivers of AMR are increasingly visible. Dalisay et al. describe lakeshore communities in the Philippines where misconceptions about antibiotics, non-prescription access, and environmental contamination converge. Their mixed-methods work shows how food systems, water pathways, and livestock practices contribute to AMR, including the insight that “consumption of animal products from livestock treated with antimicrobial substances” is a significant contributor to resistance spread.

Novel therapeutic avenues also emerge. Zhang L. et al. demonstrate that the RAGE inhibitor azeliragon—originally developed for neurodegenerative disease—exhibits direct anti-*Streptococcus pneumoniae* activity, with MIC50 4 μg/mL and MIC90 8 μg/mL, biofilm reduction, membrane disruption, and improved survival in mouse models. Their work illustrates how host-directed or repurposed small molecules may complement traditional antibiotics.

Cao et al. present a focused retrospective analysis of 32 patients with extra-urogenital *Mycoplasma hominis* infections, showing that quinolone therapy (levofloxacin, moxifloxacin) effectively reduces inflammatory markers such as WBC and CRP, underscoring its therapeutic value for these difficult-to-manage infections. Their findings provide practical guidance for risk stratification and treatment selection in complex *M. hominis* cases.

Other contributions broaden the AMR lens beyond bacteria. Li H. et al. show that HIV/HCV co-infected patients with virological failure have lower HIV-1 drug resistance rates (37.5%) than mono-infected patients (55%), suggesting co-infection status influences resistance evolution and supporting early genotyping in co-infected populations. Alanazi et al.'s meta-analysis on MDR-TB in Saudi Arabia identifies previous TB treatment as the strongest risk factor (OR 7.34), reinforcing the need for diagnostic stewardship and targeted prevention.

Education remains a powerful driver of change. Khudhur et al. demonstrate that school-based AMR education for teachers in Iraq significantly improves knowledge and attitudes (*p* < 0.001), positioning educators as multipliers for community-level awareness.

Finally, Zaheer's review of Pakistan synthesizes the socio-economic burden of AMR—rising resistance, fragmented surveillance, widespread non-prescription use, and substantial economic impacts on households and health systems.

## Conclusions

The editors of this Topic strongly believe that a decisive response to AMR demands coordinated investment, political will, and long-term vision. Countries must invest in integrated surveillance that links hospital reporting, genomic sequencing, and environmental monitoring. Early detection only works when data move rapidly across institutions—and publishing genomic data years after strain isolation makes it far less actionable. Practical stewardship must be expanded across human and animal health systems, not as theoretical instruction but as hands-on, accountable practice in hospitals, pharmacies, and veterinary supply chains. One Health actions need real authority to coordinate regulation, farm sanitation, and water-safety interventions where human, animal, and environmental risks converge. High-impact settings such as ICUs and agricultural water systems must be prioritized, as improvements here translate immediately into lives saved and resistance prevented. Finally, global progress depends on equitable access to tools—sequencing, diagnostics, and stewardship resources must be made available to all low- and middle-income countries.

From our work in Mexico, two realities stand out. First, referral bias hides the true burden: national labs with sequencing capacity often receive only the most resistant isolates, and are able to deeply and timely analyze even less, masking community-level trends. Second, water and wastewater systems remain overlooked drivers of AMR spread in rapidly growing urban regions, as do animals in agricultural communities. Addressing these gaps—in Mexico and across Latin America—is essential if we are to slow resistance before it becomes unmanageable.

The studies in this Topic show that there is hope. AMR can be mitigated through better detection, smarter stewardship, and coordinated policy. For this to become reality, it requires local and global funding, political will, and the practical integration of science into everyday clinical, agricultural, and market practices. If we act now, we can bend the curve of resistance and protect the medicines on which modern health depends.

